# Decoding the dynamics of dental distributions: insights from shark demography and dispersal

**DOI:** 10.1098/rspb.2022.0808

**Published:** 2022-06-29

**Authors:** Sora L. Kim, Justin D. Yeakel, Meghan A. Balk, Jaelyn J. Eberle, Sarah Zeichner, Dina Fieman, Jürgen Kriwet

**Affiliations:** ^1^ School of Natural Science, University of California Merced, Merced, CA, USA; ^2^ Department of Geophysical Sciences, University of Chicago, Chicago, IL, USA; ^3^ Paleobiology, National Ecological Observatory Network, Boulder, CO, USA; ^4^ Department of Geological Sciences and Museum of Natural History, University of Colorado, Boulder, CO, USA; ^5^ Division of Geological and Planetary Sciences, California Institute of Technology, Pasadena, CA, USA; ^6^ School of Geography, Environment, and Earth Sciences, Victoria University of Wellington, Wellington, New Zealand; ^7^ Department of Paleontology, University of Vienna, Vienna, Austria

**Keywords:** metapopulation, Gulf of Mexico, Arctic, Antarctic, Delaware Bay, latitudinal gradient

## Abstract

Shark teeth are one of the most abundant vertebrate fossils, and because tooth size generally correlates with body size, their accumulations document the size structure of populations. Understanding how ecological and environmental processes influence size structure, and how this extends to influence these dental distributions, may offer a window into the ecological and environmental dynamics of past and present shark populations. Here, we examine the dental distributions of sand tigers, including extant *Carcharias taurus* and extinct *Striatolamia macrota*, to reconstruct the size structure for a contemporary locality and four Eocene localities. We compare empirical distributions against expectations from a population simulation to gain insight into potential governing ecological processes. Specifically, we investigate the influence of dispersal flexibility to and from protected nurseries. We show that changing the flexibility of initial dispersal of juveniles from the nursery and annual migration of adults to the nursery explains a large amount of dental distribution variability. Our framework predicts dispersal strategies of an extant sand tiger population, and supports nurseries as important components of sand tiger life history in both extant and Eocene populations. These results suggest nursery protection may be vital for shark conservation with increasing anthropogenic impacts and climate change.

## 1. Introduction

Sharks have been a cornerstone of oceanic communities for hundreds of millions of years, a rare constant in a sea of change. The enormous spatial and temporal dominance of shark species suggests considerable ecological plasticity, which has likely contributed to their evolutionary success and may be key to understanding the ongoing and future effects of climate change on this diverse group [1]. Documenting the success of sharks as marine predators has followed a trail of fossilized teeth, accumulating in ocean sediments and indirectly recording their ecological variability and the oceanic conditions in which they lived. While fossil shark teeth assemblages have been used to elucidate water temperature and salinity [2] as well as species’ age distributions [3], ontogenetic stages [4] and the presence of nurseries [5,6], the ecological mechanisms driving population size structure remain enigmatic even in extant populations. Given that tooth size scales allometrically with body size [7], accumulations of shark teeth within narrow temporal windows may provide insight into the functioning of shark populations and communities.

Body size has an enormous influence on the structure and functioning of marine communities [8,9]. Following birth, individuals must acquire enough energy to both build and maintain somatic tissue, achieving reproductive maturity and eventually reaching an asymptotic body size [10]. Because the majority of shark species are ectothermic, the rate at which individuals grow is constrained not only by resource availability [11] but also by water temperature [12]. As temperature varies seasonally and spatially, shark species that migrate between regions are subject to changing growth rates as they transition from juvenile to adult size classes [13]. Further, some species may integrate behaviours that take advantage of differentials in resources and temperature to escape smaller body sizes more quickly. For example, many contemporary shark species give birth in warmer estuarine environments where resources are plentiful and large predators are rare, whereupon individuals migrate to colder pelagic environments as they grow [14]. Such life-history processes, especially those contributing to dispersal over time and space, will imprint on the dental distributions left behind, and may be one of the few windows into the ecologies of ancient shark species and their relationships to past climates.

**Figure 1.**
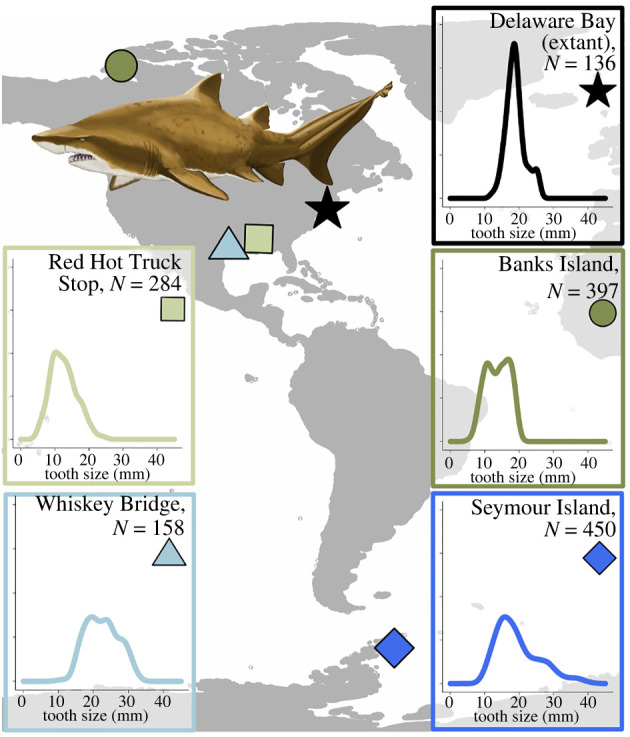
Map of sand tiger localities and size distributions based on anterior tooth crown height. In the Eocene, ${\;}^{†}$*Striatolamia macrota* inhabited high latitude waters (darker shades), such as Banks Island, Canada (Eureka Sound Fm., circle) and Seymour Island, Antarctica (La Meseta Fm., diamond), as well as mid-latitude waters (lighter shades) in the Gulf of Mexico, such as the Red Hot Truck Stop, Mississippi (Tuscahoma Fm., square) and Whiskey Bridge, Texas (Crockett Fm., triangle). These sites also represent brackish (Banks Island, high latitude, and Red Hot Truck Stop, low latitude; greens) and marine (Seymour Island, high latitude, and Whiskey Bridge, low latitude; blues) waters. Extant sand tigers were caught at Delaware Bay (star) and anterior tooth crown height is transformed from total length. ${\;}^{†}$*Striatolamia macrota* illustration by Christina Spence Morgan. (Online version in colour.)

The Eocene Epoch (56–33.9 Ma) is known for its abundant shark fossil record, with locations that ranged from the equator to both poles [1,15–17]. This time period may represent a deep-time analogue for the current climate crisis [18], perhaps facilitating a better understanding of how contemporary shark species might respond to similar environmental pressures. Extant sand tigers (*Carcharias taurus*, Lamniformes: Odontaspididae) grow up to *ca* 300 cm in length and occupy lower-latitude continental margins around the world, where they primarily hunt for prey along the sea bottom. During the Eocene, sand tigers (${\;}^{†}$*Striatolamia macrota*, Agassiz, 1843 and ${\;}^{†}$*Carcharias macrota*, Odontaspididae; extinct species denoted with${\;}^{†}$) occupied a nearly continuous latitudinal gradient ranging from the Arctic to Southern Ocean, demonstrating their remarkable plasticity. While prevalent today, the sole evidence of the vast geographical distribution and evolutionary success of sand tigers in the past is contained within local collections of fossilized teeth. For example, high-latitude sites (figure 1, darker shades) such as Banks Island were deltaic, brackish zones in the Canadian Arctic with reduced salinity [2,19] and low shark diversity [20] (figure 1, circle), whereas sites such as Seymour Island off the Antarctic Peninsula were fully marine habitats [21] with high shark diversity [16] (figure 1, diamond). Despite these environmental differences, sand tigers occupied both locales during the Eocene [16,20], in addition to lower latitude environments, notably in the Gulf of Mexico [17] (figure 1, lighter shades). Low latitude Eocene sites exhibit an environmental gradient similar to that of the high latitude sites, but in warmer waters with less seasonal variability. For example, the low latitude Red Hot Truck Stop locality of the Tuscahoma formation (Fm) in Mississippi was a reduced salinity habitat [22] (figure 1, square), similar to that of Banks Island in the Arctic, whereas the Whiskey Bridge locality of the Crockett Fm. in Texas reflected a more diverse assemblage characteristic of pelagic communities [17] (figure 1, triangle), bearing greater similarity with Seymour Island in the Southern Ocean. Compellingly, these dental distributions reveal unique and idiosyncratic attributes, which may encode important ecological relationships governing Eocene sand tiger populations.

**Figure 2.**
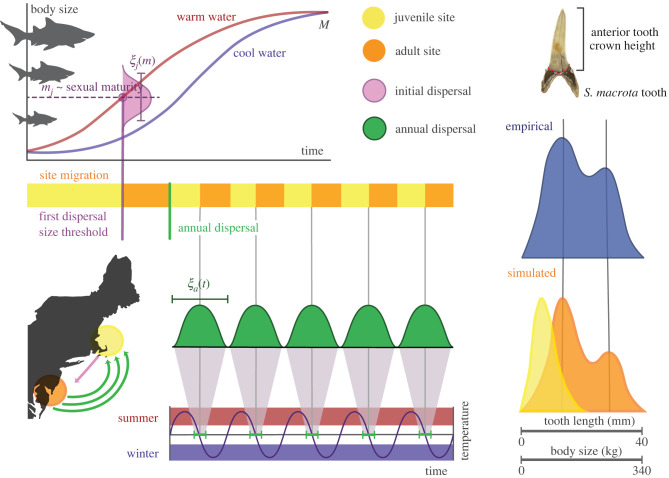
Conceptual diagram of the population simulation where sand tiger individuals migrate between a juvenile/nursery (yellow) and adult site (orange). The ontogenetic growth rates of sand tiger individuals increase with temperature (blue and red growth curves) from an initial mass *m*_0_ to an asymptotic mass *M*. After birth, newborns reside in the juvenile site until they reach maturity at mass *m*_*j*_, after which they disperse to the adult site. The juvenile dispersal window *ξ*_*j*_(*m*) (pink) denotes the variability in size at which initial dispersal occurs. Adults disperse to the juvenile site, where *ξ*_*a*_(*t*) denotes the variability in the timing of the migration (green), which occurs annually from the adult to the juvenile site and back (map inset). Individuals drop teeth as they migrate, such that accumulating dental distributions capture the size structure of populations at both sites. Empirical dental distributions (blue distribution) can be compared to simulated distributions at juvenile and adult sites (yellow and orange distributions, respectively) and evaluated for best-fit based on mean, variance, and modality, thereby gaining insight into life-history characteristics such as the juvenile and adult dispersal windows. Illustration by Christina Spence Morgan. (Online version in colour.)

To what extent can life-history dynamics drive accumulations of sand tiger teeth, and is it possible to infer such processes from the distributions themselves? Here, we examine sand tiger dental distributions from four Eocene localities that span high- and low-latitudes, as well as a contemporary sand tiger population near Delaware Bay (figure 1, star). We observe that shark dental distributions vary not only in terms of means and variance, but that some reveal pronounced bimodality while others do not. We then assess how temperature, seasonality, and dispersal to and from a nursery, or juvenile, site can affect the shapes of dental distributions. To investigate these effects, we constructed a mechanistic model of a two-site shark metapopulation, where one site serves as a coastal nursery (the juvenile site) and the other serves as a pelagic adult habitat (the adult site; figure 2). Although our model has many generalizations and assumptions, this framework correctly predicts the size structure of a contemporary sand tiger population, as well as aspects of known life-history traits characterizing the dispersal habits of sand tigers occupying the Delaware Bay. Application of our approach to sand tiger populations emphasizes the importance of seasonal adult dispersal as well as the role of juvenile sites serving as nursery localities from the Eocene to the present in both high- and low-latitude localities. That our results support the presence of shark nurseries across a range of oceanic conditions spanning 50 Myr lends particular credence to the notion that protecting nurseries may be vital for shark conservation in the face of future climate change.

## 2. Methods

### (a) Tooth identification and measurement

Shark species in the fossil record are largely identified by their tooth morphology [23] due to the poor preservation of cartilaginous skeletons. ${\;}^{†}$*Striatolamia macrota* teeth are identified by emphasized striations on the lingual side relative to the smooth labial side [23]. Anterior teeth (A1-2 and a1-2) are recognized by their long and narrow shape and acute angle between two roots, compared to the lateral and posterior teeth that have a short, blade-like appearance [20,23,24]. This tooth position was chosen as a proxy for body size due to its large size and distinct morphology compared to other tooth positions within the jaw (upper right, figure 2). Limiting the positions measured from fossil teeth prevents potential for over representation of a single individual within the assemblage. We measured anterior tooth height from the enameloid base to the blade tip of the labial side with digital calipers to an accuracy of 0.1 mm. Every seventh tooth was re-measured for 0.3 mm accuracy. The modern analogue for the Eocene ${\;}^{†}$*S. macrota* is the extant sand tiger *C. taurus* based on the similarities in tooth shape throughout the entire dentition [24]. We transformed total length measurements from the 2012 tagging season in Delaware Bay [25,26] to anterior tooth crown height based on the allometric relationships from Shimada *et al.* [7] and previously applied to fossil ${\;}^{†}$*S. macrota* in Kim *et al.* [3].

${\;}^{†}$*Striatolamia macrota* teeth from Banks Island are curated at the Canadian Museum of Nature (CMN; Ottawa, Ontario Canada); teeth from Seymour Island are curated at the University of California Museum of Paleontology (UCMP; Berkeley, CA, USA), Paleontological Research Institute (PRI; Ithaca, NY, USA), and Swedish Natural History Museum (NRM; Stockholm, Sweden); teeth from Red Hot Truck Stop locality are curated at the Carnegie Museum of Natural History (CM; Pittsburgh, PA, USA); and teeth from Whiskey Bridge locality are curated at the Whiteside Museum of Natural History (WMNH; Seymour, TX, USA). Locality descriptions are provided in electronic supplementary material, appendix I.

### (b) Population simulation

To explore specific ecological mechanisms that may be responsible for the observed dental distributions, we employed a process-based model allowing us to incorporate likely physiological and ecological constraints influencing shark populations. We constructed a two-site size-class model that tracks female shark populations over time, where one of the two sites is designated a juvenile site, or nursery, and the other is designated an adult site (figure 2). Because there is dispersal from the juvenile to adult site, and from the adult to juvenile site, each locality hosts a complex size-structure formed from a mixture of younger and older shark individuals, and it is this mixture from which accumulated tooth distributions are derived. As there is not significant sexual dimorphism among sand tigers [27], our model considers only the population dynamics of females. See electronic supplementary material, appendix II for a detailed description of the population simulation.

We considered four key dynamics influencing changes in population size for both sites: reproduction, somatic growth, mortality and dispersal between sites. In our framework, reproduction takes place only at the juvenile site, whereas mortality occurs at both sites. The *per capita* reproductive rate *r* was thus set to *r* = 0 at the adult site, and $r=0.47\times 10^{-7}\;\mathrm{female}\;\mathrm{inds}/s$ [28] at the juvenile site, independent from time of year or water temperature. The *per capita* mortality rate was assumed to be constant across size classes within both juvenile and adult sites at $\mu =5.71\times 10^{-9}\;\mathrm{inds}/s$ [12]. A constant mortality rate has been successfully applied to models of contemporary shark populations [12] and is more parsimonious than assuming a particular survivorship curve, particularly for Eocene systems. Shark individuals follow size-dependent growth rates, increasing in mass *m* (g) following the growth trajectory described by West *et al.* [10] as a function of temperature-dependent metabolic rate (see illustration of ontogenetic trajectories in cold and warm environments in figure 2 and electronic supplementary material, appendix II for details). Accordingly, shark individuals grow more quickly in warm environments, reaching the asymptotic mass *M* at a younger age.

In our two-site model, juveniles disperse to the adult site once they have reached a particular mass, and adult females migrate annually from the adult to juvenile site to reproduce. The initial dispersal of juveniles to the adult site and annual dispersal of adults to the juvenile site are considered separately because we assume these events are mass-dependent and time-dependent, respectively. As offspring grow in size to maturation *m*_*j*_, their migration rate to the adult site increases sigmoidally. The juvenile dispersal window *ξ*_*j*_ describes the flexibility of this mass threshold: a smaller dispersal window (low *ξ*_*j*_) means that initial dispersal of juveniles to the adult site operates around a strict mass threshold *m*_*j*_, whereas a large juvenile dispersal window (high *ξ*_*j*_) means that initial dispersal of juveniles to the adult site is flexible around *m*_*j*_.

We assume that individuals occupying the adult site disperse back to the juvenile site to reproduce annually, such that the adult dispersal rate is a function of time. The adult dispersal window *ξ*_*a*_ describes the flexibility of this annual dispersal: a smaller dispersal window (low *ξ*_*a*_) means that annual adult dispersal to the juvenile site operates around a strict peak day, whereas a large adult dispersal window (high *ξ*_*a*_) means that annual adult dispersal to the juvenile site is flexible. We note that the resolution and range of juvenile and adult dispersal windows had to be adjusted from site to site to account for simulation limitations related to population dynamics in different temperature environments. As individuals grow and disperse over time, they drop teeth at a constant rate [29], such that the accumulation of differently sized dentition mirrors the size-distribution of sharks visiting each site. While tooth shedding rates can vary seasonally [30,31], the inclusion of this dynamic has very little effect on model results (electronic supplementary material, appendix III).

### (c) Comparing observed and simulated dental distributions

Our overall goal is to use the known conditions generating simulated dental distributions to gain insight into the unknown conditions generating empirical dental distributions. Specifically, we aim to evaluate (i) whether an observed distribution is better described as a juvenile versus adult site and (ii) which dispersal strategy—strict versus flexible juvenile and adult dispersal windows—may have contributed to the observed distributional geometry. To compare simulated dental distributions to those observed from contemporary and Eocene localities, we first parameterized the model with estimated winter minimum and summer maximum mean ocean temperatures for both juvenile and adult sites. See electronic supplementary material, appendix III for site temperature estimates. We then simulated dental distributions for both juvenile and adult sites across all combinations of (*ξ*_*j*_, *ξ*_*a*_). Because simulated distributions were both non-normal and multi-modal, to compare distributions we constructed a single error term taking into account seven shape parameters, including the first two moments, the presence/absence and numerically estimated values of one or multiple modes, and the 25th, 50th (median) and 75th percentiles. Error was then calculated as

2.1 $$\epsilon_{\;j,a}(\xi_{j},\xi_{a})=\sum_{k=1}^{7}|w_{k}^{\mathrm{sim}}(\xi_{j},\xi_{a})-w_{k}^{\mathrm{obs}}|/w_{k}^{\mathrm{obs}},$$

for juvenile and adult sites, where $w_{k}^{\mathrm{sim}}(\xi_{j},\xi_{a})$ and $w_{k}^{\mathrm{obs}}$ are the measured values for the shape parameters described above with respect to simulated and observed tooth distributions respectively, given the simulated dispersal windows (*ξ*_*j*_, *ξ*_*a*_). Accordingly, the simulated juvenile or adult site dental distribution with a lower *ε* value will indicate a better match for the observed dental distribution, and the particular combination of (*ξ*_*j*_, *ξ*_*a*_) that results in the lower *ε* value will point to the best-fit dispersal strategy.

## 3. Results and discussion

### (a) Discerning distributions of dentition

The life history and movement of extant sand tigers (*Carcharias taurus*) has been examined extensively in the western Atlantic, in particular for the population near Delaware Bay (figure 1, star). A long-standing tagging programme, led by Delaware State University and University of Delaware between 2007 and 2015, recorded biological information such as fork length, total length, sex and maturity state, as well as annual/seasonal movement data via acoustic and satellite tagging [25,26]. Occupation of the Delaware Bay by sand tigers correlates strongly with temperature, with juveniles arriving approximately one month prior to adults [25,26]. The residence time for the majority of individuals is of the order of 150 days and sharks disperse from the site once the temperatures decrease in October [25,26]. The dental distribution for the contemporary Delaware Bay locality was reconstructed from allometric relationships between tooth size and length [7], with measures of length from 136 individuals [25,26] giving a mean ± s.d. crown height of 18.92 ± 2.88 mm (median = 18.63 mm). The maximum tooth length observed was estimated to be 26.11 mm, corresponding to a total body length of 295 cm. Previous work based on 96 sand tiger individuals revealed the asymptotic body length for this species to be 296 cm [27], providing support for the tooth height/body length conversion.

The Eocene sites explored here are well-known and iconic in the palaeontological literature. The Eureka Sound Fm. on Banks Island (Canada, figure 1, circle) and La Meseta Fm. on Seymour Island (Antarctica, figure 1, diamond) are the most fossiliferous high latitude Eocene sites, with previous studies focused on sedimentology, flora, and fauna [32,33]. In both sites, the extinct sand tigers are well-represented by ${\;}^{†}$*S. macrota* and another ${\;}^{†}$*Carcharias* species [16,20]. The geology of the Eureka Sound Fm. on Banks Island (Canada) points to a coastal deltaic environment with low shark diversity during the Eocene [20], whereas the La Meseta Fm. on Seymour Island (Antarctica) is noted for its rich and diverse marine assemblage that includes 35 species of sharks [16]. Among low-latitude Eocene sites, the Tuscahoma Fm. at the Red Hot Truck Stop (MS) is largely known for its mammalian record, and is preserved in a lithology that suggests a large-scale, fluvial-dominated deltaic system [22] (figure 1, square). Fully marine habitats are rare across the Eocene Gulf of Mexico, however the Crockett Fm. at Whiskey Bridge (TX) is one of the most fossiliferous Eocene marine sites known [34] (figure 1, triangle). While the Red Hot Truck Stop and Whiskey Bridge localities are relatively proximal along the Gulf, they are not necessarily contemporaneous as the Tuscahoma Fm. spans the Palaeocene–Eocene Thermal Maximum [22] whereas the Crockett Fm. is Middle Eocene [34], and likely represent distinct sand tiger populations.

The large sample sizes of sand tiger teeth from the Eocene sites allows for population-level analyses in the fossil record, which is rare for vertebrate fossil assemblages. We measured a total of 1053 anterior fossil sand tiger teeth across the four fossil sites (see electronic supplementary material, table), with distinct distributional geometries characterizing each locality (figure 1). The Banks Island collection consisted of 397 anterior teeth with a mean crown height of 13.70 ± 3.41 mm (median = 14.10 mm). The Seymour Island collections consisted of 450 anterior teeth with mean crown height of 19.61 ± 6.39 mm (median = 18.00 mm). The Red Hot Truck Stop collection included 284 anterior teeth with mean crown height of 12.62 ± 3.82 mm (median = 12.10 mm). Finally, the Whiskey Bridge collection included 158 anterior teeth with mean crown height of 22.51 ± 4.59 mm (median = 22.55 mm). For each assemblage, we examined the effect of sample size on our estimation of dental distribution shape parameters using both parametric and non-parametric methods (electronic supplementary material, appendix I). While some dental distributions are distinctly non-normal, both examinations confirm that the sample sizes accurately capture lower order moments (means, standard deviations, medians; estimation accuracy within 0.2–0.5 mm), and are adequate for estimating higher-order moments such as modes (estimation accuracy within 1–2 mm).

### (b) A contemporary dental distribution predicts known dispersal strategies

**Figure 3.**
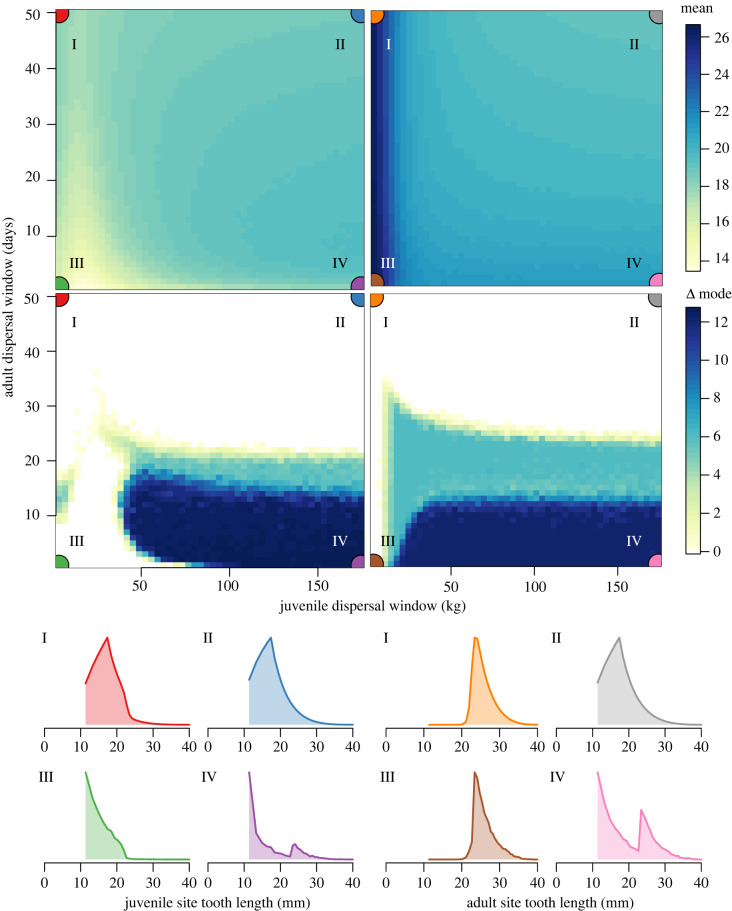
Simulation results for the dynamic population model as a function of juvenile and adult dispersal windows (*ξ*_*j*_ and *ξ*_*a*_, respectively). Changes in dental distribution shape are captured by site-specific means (top two panels) and the distance between modes (Δ mode; bottom two panels). A Δ mode value of zero means there is only one mode. Representative distributions of anterior tooth crown height are shown for juvenile site and adult sites for regions I–IV (horizontal along bottom and vertical along right edge, respectively), where colour denotes both region and site identity. Regions I–IV depict various combinations of small and large dispersal windows. Region I (high *ξ*_*a*_, low *ξ*_*j*_); II (high *ξ*_*a*_, high *ξ*_*j*_); III (low *ξ*_*a*_, low *ξ*_*j*_); IV (low *ξ*_*a*_, high *ξ*_*j*_). Results are shown for high-latitude Eocene conditions, but are qualitatively similar for all evaluated localities. See electronic supplementary material, appendix IV for details and results for contemporary and low-latitude Eocene conditions. (Online version in colour.)

The results of our population simulation reveal that changes in the initial dispersal of younger sharks from the juvenile site to the adult site, and of older sharks from the adult to juvenile site, can drastically change the shape of dental distributions within both sites (figure 3; electronic supplementary material, appendix IV). While it is relatively straightforward to show how different life-history characteristics may influence distributions of accumulated shark teeth by forward-simulation, it is a more difficult prospect to start with a distribution and attempt to back-calculate some understanding of the potential ecological drivers from which it emerged. We next examine whether and to what extent we can gather ecological insight into dispersal strategies from a well-known contemporary sand tiger population based on our established framework.

Extant sand tiger sharks (*C. taurus*) are highly migratory along the continental shelf of the western margin of the Atlantic Ocean [25,26,35,36]. In the Delaware Bay, sand tigers aggregate in the summer to autumn, and include a mixture of both juvenile and adult size classes [25,26]. The proposed nursery for sand tigers in the western Atlantic is the Plymouth, Kingston, Duxbury Bay (MA) where individuals span 78–104 cm (ATCH range 8.3–10.4 cm) [35], substantially smaller and younger than individuals in Delaware Bay. As such, the Delaware Bay aggregate is thought to represent a mixed age population at an adult site, where dispersal to the Bay corresponds closely with seasonal temperature [26]. Acoustic tagging efforts indicate a gradual arrival of sand tigers, where juveniles begin arriving in early May with adults arriving approximately one month later [25]. In contrast to their arrival, the departure window at Delaware Bay is more tightly constrained from early to mid-October [25,26]. Most individuals are present in the Bay for *ca* 150 days, which is roughly a 40-day standard deviation around the peak migration time [25]. This well-studied modern sand tiger population at Delaware Bay thus provides a distinct opportunity to examine whether certain aspects of the well-known life histories can be disentangled from the distributions alone.

**Figure 4.**
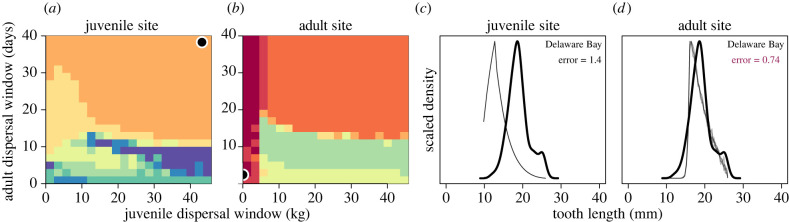
Comparison of empirical dental distributions from sand tigers in Delaware Bay and those simulated across different values of the juvenile (*ξ*_*j*_) and adult (*ξ*_*a*_) dispersal windows. Better fits between empirical and simulated distributions at juvenile (*a*) and adult sites (*b*) are represented by warmer colours (lower *ε*; equation (2.1)). Each sequential change in colour represents a 10% change in error. Best-fit simulation results for juvenile and adult simulation results at a particular (*ξ*_*j*_, *ξ*_*a*_) are denoted by black circles. The corresponding distributions at this best-fit value of (*ξ*_*j*_, *ξ*_*a*_) are shown for juvenile (*c*) and adult (*d*) sites for comparison (thin lines) relative to the empirical distribution (thick line). Within-site best-fit error values are reported in the upper-right, and the across-site best-fit error is denoted in bold. (Online version in colour.)

We systematically compared the dental distributions produced by our population simulation across values of *ξ*_*j*_ and *ξ*_*a*_ for both juvenile and adult sites against the empirical distribution from the Delaware Bay by estimating error in model fit *ε* (equation (2.1)). We compare the model fit error for both the simulated juvenile and adult sites (*ε*_*j*_ versus *ε*_*a*_) by first investigating whether our framework was capable of detecting if the Delaware Bay population more likely represented a juvenile versus adult site. Our assessment reveals that the minimal error for the juvenile site is *ε*_*j*_ = 1.4, whereas the minimal error for the adult site is *ε*_*a*_ = 0.74 (figure 4). This suggests that the Delaware Bay population represents an adult rather than juvenile population, confirming what is already understood [25,35], but more importantly—at broad strokes—validating the utility of our approach.

Except for the scenarios where dental distributions are very similar (high *ξ*_*j*_ and *ξ*_*a*_) or bimodal (high *ξ*_*j*_ and low *ξ*_*a*_), site identity is relatively straightforward to distinguish based on differences in tooth size means. A more rigorous assessment of our approach is to examine whether the best-fit parameterization of dispersal windows (*ξ*_*j*_, *ξ*_*a*_) correlates with our understanding of the Delaware Bay system. We find that, given the adult site identification of the Delaware Bay population, the best-fit dispersal window parameterization indicates a strict mass at which juveniles leave for the adult site. While the lowest absolute error also points to a strict temporal window describing adult dispersal, the lowest 10% error covers a wide range of possible values—from 1 to 40 days (dark red colour in figure 4*b*). The 40-day standard deviation in the timing of adults to Delaware Bay [25] is not inconsistent with our results, however, our error analysis shows that we cannot reliably discern specific adult dispersal window size if the juvenile dispersal window is also small (figure 4). More importantly, a small juvenile dispersal window supports an interpretation of the Plymouth, Kingston, Duxbury Bay as a nursery where pups remain until a strict size threshold is reached (low *ξ*_*j*_) before dispersing to the Delaware Bay site. We therefore suggest that our framework provides support for the role of nurseries with respect to the Delaware Bay population, which we infer based only on the shape of the dental distribution.

### (c) Deciphering life history and dispersal in the Eocene

Understanding the nature of shark communities in response to documented changes in Eocene climate may provide insight into the future of shark populations in our changing oceans. Because our primary window into these systems is through the lens of accumulated teeth, interpreting dental distributions from an ecological perspective may permit disentangling aspects of their ecologies, such as life-history mediated dispersal behaviours. We thus use our framework to examine the underlying ecological constraints potentially driving the accumulation of dental distributions.

**Figure 5.**
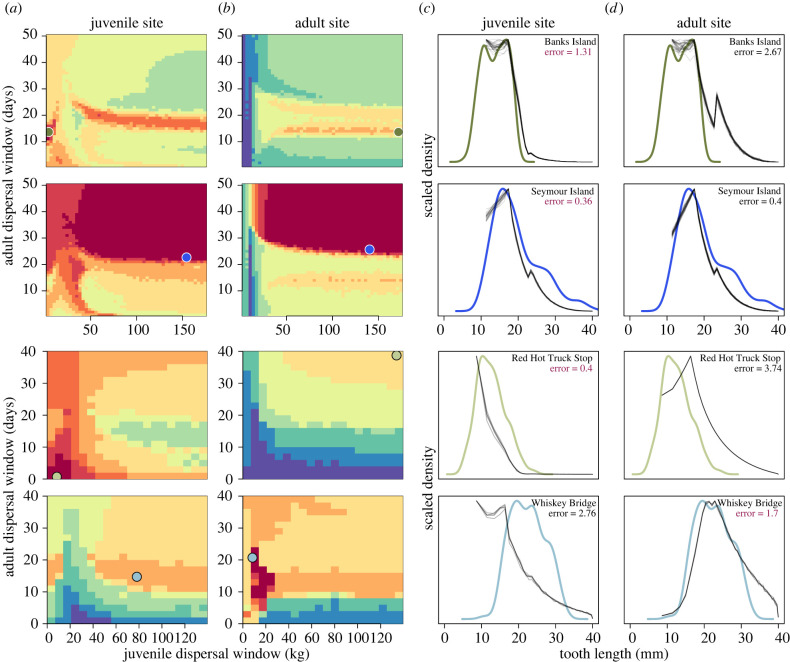
Comparison of empirical dental distributions from sand tigers at Eocene localities and those simulated across different values of the juvenile (*ξ*_*j*_) and adult (*ξ*_*a*_) dispersal windows. Eocene localities include the high latitude Banks Island (first row, dark green) and Seymour Island (second row, dark blue) sites as well as the low latitude Red Hot Truck Stop (third row, light green) and Whiskey Bridge Sites (fourth row, light blue). Blues denote sites reconstructed as marine habitats; greens denote sites reconstructed as near shore estuarine habitats. Across all rows, better fits between empirical and simulated distributions at juvenile (*a*) and adult sites (*b*) are represented by warmer colours (lower *ε*; equation (2.1)). Each sequential change in colour represents a 10% change in error. Best-fit simulation results for juvenile and adult simulation results at a particular (*ξ*_*j*_, *ξ*_*a*_) are denoted by coloured circles. The corresponding distributions at this best-fit value of (*ξ*_*j*_, *ξ*_*a*_) are shown for juvenile (*c*) and adult (*d*) sites for comparison (thin lines) relative to the empirical distribution (thick line). Within-site best-fit error values are reported in the upper-right, and the across-site best-fit error is denoted in bold. (Online version in colour.)

Our evaluation of sand tiger dental distributions from both high- and low-latitude brackish and marine sites aligns with palaeontological reconstructions of site habitat. For high-latitude locations, we observe that the Banks Island (Canada) site better fits the simulated juvenile (*ε*_*j*_ = 1.31) relative to adult site (*ε*_*a*_ = 2.67), and the Seymour Island (Antarctica) site better fits the simulated juvenile (*ε*_*j*_ = 0.36) relative to adult site (*ε*_*a*_ = 0.40), though this latter difference is negligible (rows 1–2, figure 5). The fossil shark tooth-bearing strata of Banks Island (Eureka Sound Fm.) dates to the early-middle Eocene [20], and is reconstructed to be a channel or mouth bar deposit of a delta front [37]. The presence of sand tiger teeth in unconsolidated sands, a fossilized crocodyliform fossil [38], and low palaeosalinity [2,19], all point to a mild and brackish estuarine environment [39,40], supporting our prediction of this site serving as a near shore nursery location (row 1, figure 5). By contrast, the faunal composition and geochemistry of the Seymour Island locality (La Meseta Fm.) suggest typical marine conditions [3,16,21]. This site encompasses seven stratigraphic biostratigraphy units that span middle to late Eocene; although the shark assemblage changes across the formation [16], ${\;}^{†}$*S. macrota* is the most abundant spanning *ca* 45–41 Myr and demonstrate relatively stable oceanographic conditions [3]. While our simulations narrowly support this locality serving as a juvenile site, the plateaued error surface precludes a clear interpretation, as is evident from the similarity of juvenile and adult simulated distributions (row 2, figure 5).

For low-latitude localities, we observe that the Red Hot Truck Stop (MS) better fits simulated juvenile (*ε*_*j*_ = 0.40) compared to adult sites (*ε*_*a*_ = 3.74), whereas Whiskey Bridge (TX) better fits simulated adult (*ε*_*a*_ = 1.7) compared to juvenile sites (*ε*_*j*_ = 2.76) (rows 3–4, figure 5). Palynofloral reconstruction of the Red Hot Truck Stop locality (Bashi/Tuscahoma Fm.) supports a paratropical climate [41] characterized by a large fluvial-dominated deltaic system in an estuarine habitat that spans the Palaeocene/Eocene Thermal Maximum [22]. By contrast, the Whiskey Bridge locality (Crockett Fm.) is dated to the early part of the Middle Eocene Climate Optimum [34] and represents a shallower marine habitat with normal salinity [42]. This sub-tropical climate supported at least three species of sand tigers (${\;}^{†}$*Carcharias cuspidata*, ${\;}^{†}$*C. hopei* and ${\;}^{†}$*S. macrota*) [17,42] found within the Stone City Member of the Crockett Fm. Taken together, these reconstructions support the notion that Red Hot Truck Stop and Whiskey Creek represent juvenile and adult sites, respectively, as predicted by our framework.

### (d) Dental distributions support the role of shark nurseries

The contemporary Delaware Bay sand tiger population as well as those from all Eocene sites except the ambiguous Seymour Island marine locality point to a small juvenile dispersal window, meaning that a strict size threshold initiates first dispersal to the adult site (figure 5). With regard to the Seymour Island locality, we observe that, for both simulated juvenile and adult sites, the error surface uniquely plateaus across a large range of potential (*ξ*_*j*_, *ξ*_*a*_) values providing similarly good fits (row 2 in figure 5). This error surface limits our ability to either interpret whether Seymour Island better represents a juvenile versus adult site or to confidently estimate the size of either dispersal window. However, elevated juvenile and adult dispersal windows in the Antarctic may not be surprising, as this site is known to have accumulated across a larger temporal window [43] where changing environmental conditions associated with the opening of the Drake passage [3,44] impacted shark community assembly [16], and may have influenced how sites were used by shark populations over space and time.

Contemporary shark nurseries are thought to enable resource access, promote juvenile growth, and protect vulnerable pups against mortality from potential predators [13,14]. Upon maturation and release from mesopredator pressure, dispersal to adult sites enables growing individuals access to larger prey and perhaps mating opportunities [45], though the timing of these events are variable among sand tiger populations [26,46]. In our framework, a large juvenile dispersal window means that both smaller and larger individuals initiate this first dispersal. These conditions imply that the costs and benefits of the juvenile site are similar to those of the adult site, such that it is no longer serving in the context of a nursery. By contrast, a strict juvenile dispersal window points to a sharp threshold in body size initiating first dispersal, implying that the costs and benefits characterizing juvenile and adult sites vary significantly, and supporting the notion of the juvenile site serving as a nursery.

We find strong support for strict juvenile dispersal windows, and by extension the role of juvenile sites serving as nurseries, for the contemporary sand tiger population as well as three of four Eocene localities. Declines in nursery habitats have been invoked as a potential extinction mechanism for both fossil [5] and contemporary shark populations [13,14], though the extent to which nurseries may buffer against population declines is controversial [47]. Our results reinforce the notion that nurseries have played an important role in structuring sand tiger life histories across a range of oceanic conditions spanning tens of millions of years.

## 4. Conclusion

We have shown that two important drivers of shark life history—the variability in the size of juveniles first leaving a nursery and the temporal variability marking annual migrations of adults back to a nursery—can result in the diversity of dental distributions characterizing both observed contemporary and palaeontological sand tiger populations. Our perspective is based on the correlation between the known drivers governing a mechanistic model and the unknown drivers governing empirical distributions of shark teeth. Our model demonstrates that differences in juvenile and adult dispersal strategies driven by size and seasonality can account for the observed variation in empirical distributions. Further, the inferences we draw predict expectations from contemporary observations and palaeontological reconstructions. However, our mechanistic model is necessarily limited and so the interpretations that we draw from this comparison must be carefully aligned with the underlying assumptions. While the simplistic model we outline here is meant to be a starting point in modelling ecological processes governing shark dynamics and we admittedly do not test the contribution of alternative mechanisms, future models can specify parameters based on modern shark biology and sedimentological processes.

Fossilized dental distributions are likely influenced by many biological, geological and taphonomic processes. The diverse distributions observed across shark species may emerge from differences in survivorship, sexual dimorphism and species’ position within the marine food web. Because fossil teeth are accumulated over long periods of time, differences in some dental distributions may also reflect oceanic conditions and sediment formation, taphonomic dynamics, evolutionary change and/or tectonic processes [1]. Disentangling the potential drivers of particular dental distributions must be justified from an understanding of both the geological characteristics of the locality as well as a biological understanding of the species. Our approach, while focused on one set of very influential life-history characteristics, may be well-positioned to address such alternative drivers in future efforts. Deciphering the mechanisms from which dental distributions are formed permits an ecological window into both extinct and extant shark communities, and we suggest this may generate new insights into how these enduring and enigmatic species persist in the face of change.

## Data Availability

Raw data for empirical tooth distributions are provided in the Dryad repository at https://doi.org/10.6071/M3RT05. Simulation code is available in the public GitHub repository https://github.com/jdyeakel/sharks_bodysize. The data are provided in electronic supplementary material [48].
